# Metabolism of IMM-H004 and Its Pharmacokinetic-Pharmacodynamic Analysis in Cerebral Ischemia/Reperfusion Injured Rats

**DOI:** 10.3389/fphar.2019.00631

**Published:** 2019-06-13

**Authors:** Ziqian Zhang, Dandan Liu, Jianwei Jiang, Xiuyun Song, Xiaowen Zou, Shifeng Chu, Kebo Xie, Jungui Dai, Naihong Chen, Li Sheng, Yan Li

**Affiliations:** ^1^State Key Laboratory of Bioactive Substance and Function of Natural Medicines, Beijing Laboratory of Non-Clinical Drug Metabolism and PK/PD Study, Key Laboratory of Active Substances Discovery and Drug Ability Evaluation, State Key Laboratory of Bioactive Substance and Function of Natural Medicines, Department of Drug Metabolism, Institute of Materia Medica, Chinese Academy of Medical Sciences and Peking Union Medical College, Beijing, China; ^2^State Key Laboratory of Bioactive Substances and Function Natural Medicines, Department of Pharmacology, Institute of Materia Medica, Chinese Academy of Medical Sciences and Peking Union Medical College, Beijing, China; ^3^Tianjin University of Traditional Chinese Medicine, Tianjin, China; ^4^State Key Laboratory of Bioactive Substance and Function of Natural Medicines, Institute of Materia Medica, Chinese Academy of Medical Sciences and Peking Union Medical College, Beijing, China

**Keywords:** drug metabolism, pharmacokinetic/pharmacodynamics (PK/PD), UDP-glucuronosyltransferases, cytochromes P450, cerebral ischemia, neuroprotection, IMM-H004

## Abstract

IMM-H004, a derivative of coumarin, is a promising candidate for the treatment of cerebral ischemia. The pharmacodynamic mechanisms of IMM-H004 are still under exploration. The present study was conducted to explore the pharmacoactive substances of IMM-H004 from the perspective of drug metabolism. Four metabolites of IMM-H004 including demethylated metabolites M1 and M2, glucuronide conjugate IMM-H004G (M3), and sulfated conjugate M4 were found in rats *in vivo*. IMM-H004G was the major metabolite in rats and cultured human hepatocytes, and uridine diphosphate-glucuronosyltransferase (UGT) was found to catalyze the metabolism of IMM-H004 in human liver microsomes (HLMs) and rat liver microsomes (RLMs) with high capacity (*V*_max_ at 3.25 and 5.04 nmol/min/mg protein). Among 13 recombinant human UGT isoforms, UGT1A7, 1A9, 1A8, and 1A1 appeared to be primarily responsible for IMM-H004G formation. The exposure and duration of IMM-H004G (28,948 h × ng/ml of area under the plasma concentration–time curve (AUC), 6.61 h of *t*_1/2β_) was much higher than that of the parent drug (1,638 h × ng/ml of AUC, 0.42 h of *t*_1/2β_) in transient middle cerebral artery occlusion/reperfusion (MCAO/R) rats, consistent with the malondialdehyde (MDA) inhibition effect for at least 10 h. Further pharmacological study revealed that IMM-H004G exhibited a similar neuroprotective activity to that of the parent drug on both oxygen-glucose deprivation injured PC12 cells and transient MCAO/R injured rats. These results demonstrate that both prototype and IMM-H004G are the active pharmaceutical substances, and IMM-H004G, at least in part, contributes to the maintenance of anti-cerebral ischemia efficacy of IMM-H004.

## Introduction

Stroke is one of the leading causes of disability and death worldwide (Meschia et al., 2014). According to the World Health Organization, 15 million people suffer stroke worldwide each year. Of these, 5 million die and another 5 million are permanently disabled. About 87% of all strokes are ischemic stroke. Up to now the only drug that has been approved by the Food and Drug Administration for the treatment of ischemic stroke is the thrombolytic tissue-plasminogen activator. However, due to the short-term treatment time window and hemorrhage transformation, only a few patients benefit from the tissue-plasminogen activator (Peisker et al., 2017). Therefore, it is essential to develop other therapies for patients with acute ischemic stroke. In addition to thrombolytic, neuroprotection is considered as another strategy for the treatment of stroke (Neuhaus et al., 2017). The approval of edaravone for treating stroke patients brings the hope for the development of novel neuroprotective agent (Lee and Xiang, 2018).

Coumarins are widely distributed in a variety of plants. It was reported that natural coumarin compounds, such as umbelliferone and esculetin, had neuroprotective effects by their antioxidant and anti-inflammatory activities (Subramaniam and Ellis, 2013; Wang et al., 2015; Sulakhiya et al., 2016). In previous studies, the structure-activity relationships of a series of 3-piperazine substituted coumarin derivatives were analyzed (Li et al., 2010). It was found that IMM-H004, 7-hydroxy-5-methoxy-4-methyl-3-(4-methylpiperazin-1-yl)-coumarin, exhibited potent neuroprotective effect *in vitro* and *in vivo* (Song et al., 2013; Ji et al., 2014; Song et al., 2014; Zuo et al., 2014; Zuo et al., 2015; Chu et al., 2017; Niu et al., 2017; Peña et al., 2017). And the neuroprotective effect of IMM-H004 on cerebral ischemia rats was even better than that of edaravone (Chu et al., 2017; Niu et al., 2017). After systematical pharmacological studies, IMM-H004 was considered to be an attractive anti-cerebral ischemia drug candidate.

Despite intensive investigations into its pharmacological activities and mechanisms, the biotransformation of IMM-H004 has not been addressed. The identification of drug metabolic pathways and the characterization of the enzymes involved in drug metabolism are important aspects in drug discovery and development. Detecting and characterizing metabolites in both experimental animals and humans are not only critical to evaluate potential risks for drug development but also helpful to understand the mechanism of drug action and identify pharmacokinetic (PK) properties to further improve and optimize the compound design. Therefore, the objectives of the present study were 1) to identify the major metabolites of IMM-H004 and the enzymes responsible for IMM-H004 metabolism *in vivo* and *in vitro*; and (2) to evaluate the pharmacokinetic/pharmacodynamics (PK/PD) relationship of IMM-H004 and the neuroprotective activities of major metabolites *in vivo* and *in vitro*.

## Materials and Methods

### Chemicals and Reagents

IMM-H004 (purity >99%), M1, and M2 (purity >90%) (Li et al., 2010) were synthesized by Laboratory of Chemical Synthesis, and IMM-H004G (M3) (purity >99%) for animal study was provided by Prof. Dai (Laboratory of Biosynthesis of Natural Products, Institute of Materia Medica, Chinese Academy of Medical Science and Peking Union Medical College, Beijing, China). Propranolol (internal standard, IS), β-glucuronidase, sulfatase, 3′-phosphoadenosine-5′-phosphosulfate (PAPS), uridine 5′-diphosphoglucuronic acid (UDPGA), and alamethicin were obtained from Sigma-Aldrich (St. Louis, MO). Rat liver microsomes (RLMs) and cytosols were prepared by differential ultracentrifugation, and the protein concentration was determined by bicinchoninic acid assay (Beyotime Institute of Biotechnology, Jiangsu, China). Pooled mixed-gender human liver microsomes (HLMs), human liver cytosols, recombinant human cytochrome P450 enzymes (CYP1A1, CYP1A2, CYP2A6, CYP2B6, CYP2C8, CYP2C9, CYP2C19, CYP2D6, CYP2E1, CYP2J2, CYP3A4, CYP4A11, CYP4F2, and CYP4F3), recombinant human UDP-glucuronosyltransferases (UGT1A1, UGT1A3, UGT1A4, UGT1A6, UGT1A7, UGT1A8, UGT1A9, UGT1A10, UGT12B4, UGT12B7, UGT12B10, UGT12B15, and UGT12B17), and recombinant human sulfotransferase (SULT1A1, SULT1A2, SULT1A3, SULT1B1, SULT1C2, SULT1C4, SULT1E1, and SULT2A1) were purchased from BD Gentest (Woburn, MA).

### Animals

Male Sprague–Dawley rats (260–280 g) were purchased from Vital River Experimental Animal Co., Ltd (Beijing, China). Standard pelleted laboratory chow and water were allowed *ad libitum*. All experiments were approved by the Animal Care and Welfare Committee of Peking Union Medical College and were strictly taken in accordance with guidelines regarding the use and care of laboratory animals issued by the Institute Animal Care and Welfare Committee.

### Identification of IMM-H004

#### Metabolites in Rats

After intravenous (iv) injection with IMM-H004 citrate (6 mg/kg, dissolved in saline to yield a concentration of 1.2 mg/ml), rats were housed individually in metabolic cages to allow separate collection of urine and feces until 72 h postdose. Additional rats were bile cannulated under light ether anesthesia. Each rat was housed individually in a metabolic cage and allowed to recover from anesthesia for 2 to 3 h. Then bile duct cannulated rats were iv injected with IMM-H004 citrate (6 mg/kg, dissolved in saline to yield a concentration of 1.2 mg/ml), bile samples were collected until 24 h postdose.

β-glucuronidase and sulfatase were dissolved in physiological saline to 3 and 10 mg/ml, respectively. Urine and bile samples (50 μL) were mixed with β-glucuronidase or sulfatase solution (200 μL) and incubated at 37°C for 1 h. The incubations were quenched with two volumes of ice-cold acetonitrile. Samples without β-glucuronidase or sulfatase were used as controls. The mixtures were centrifuged at 18,800×*g* for 5 min. A 1 μL aliquot of supernatant was injected into liquid chromatography tandem mass spectrometry (LC-MS/MS) for analysis.

Feces samples were homogenized in 10-fold solvent of water and methanol (1:1) and diluted 10-fold in water. Bile and urine samples were diluted 100-fold in water. The concentrations of IMM-H004, M1, M2, and IMM-H004G in diluted bile, urine, and feces samples were determined by LC-MS/MS.

#### *In Vitro* Incubations

Cytochrome P450 (CYP450)-mediated metabolism of IMM-H004 was conducted in RLMs or HLMs. IMM-H004 (10 μM) was incubated with RLMs/HLMs (0.5 mg protein/ml) in a final volume of 0.2 ml Tris-HCl buffer (50 mM, pH 7.4) containing 5 mM MgCl_2_. After 2 min preincubation at 37°C, the reactions were initiated by the addition of reduced nicotinamide adenine dinucleotide phosphate (NADPH) regeneration system (10 mM β-nicotinamide adenine dinucleotide phosphate, 100 mM glucose-6-phosphate and 10 U/ml 6-G-P dehydrogenase). After incubation for 30 min, the reactions were terminated by adding two volumes of ice-cold acetonitrile. Samples without NADPH were used as controls. The incubation mixture was vortexed and centrifuged at 18,800×*g* for 5 min. A 5 μL aliquot of the supernatant was injected into LC-MS/MS for analysis.

Glucuronidation reactions were characterized in RLMs or HLMs. The glucuronidation incubation mixture contained IMM-H004 (10 μM), RLMs/HLMs (1 mg protein/ml), alamethicin (50 μg/mg protein), and UDPGA (3 mM) in Tris-HCl buffer (50 mM, pH 7.4) containing 5 mM MgCl_2_ at a final volume of 200 μL. After preincubation on ice for 15 min, the reactions were initiated by the addition of UDPGA and were incubated at 37°C for 30 min before being quenched with two volumes of ice-cold acetonitrile. Samples without UDPGA were used as controls. The incubation mixture was vortexed and centrifuged at 18,800×*g* for 5 min. A 1 μL aliquot of the supernatant was injected into LC-MS/MS for analysis.

Sulfation reactions were carried out using rat or human liver cytosol. The incubation mixture contained IMM-H004 (10 μM), rat/human liver cytosol (1 mg protein/ml), and PAPS (3 mM) in potassium phosphate buffer (100 mM, pH 7.4) at a final volume of 200 μL. After 5 min preincubation at 37°C, the reactions were initiated by the addition of PAPS. After incubation for 30 min, the reactions were terminated by the addition of two volumes of ice-cold acetonitrile. Samples without PAPS were used as controls. The incubation mixture was vortexed and centrifuged at 18,800×*g* for 5 min. A 1 μL aliquot of the supernatant was injected into LC-MS/MS for analysis.

#### Structural Identification of Metabolites by Liquid Chromatography Tandem Mass Spectrometry and Nuclear Magnetic Resonance (NMR)

Identification of M1 and M2 was based on comparing the retention time and fragmentation mass spectrum with standards. Chromatographic separation was performed on a CAPCELL PAK ADME (absorption, distribution, metabolism, and excretion) column (3 μm, 2.1 mm × 100 mm, Shiseido, Tokyo, Japan). The mobile phases were water with 0.5% formic acid (mobile phase A) and methanol with 0.5% formic acid (mobile phase B) pumped at 0.25 ml/min. The elution condition was 20% mobile phase B for 1.0 min, ascending to 80% B in 8.0 min, holding for 1.0 min, and reequilibrating to 20% B within 0.5 min and maintained for 3.5 min.

IMM-H004G was separated on a Zorbax C18 column (5 μm, 4.6 mm × 150 mm, Agilent, USA). The mobile phases were water with 0.5% formic acid (mobile phase A) and acetonitrile with 0.5% formic acid (mobile phase B) pumped at 1 ml/min. The elution condition was started with 2% mobile phase B, ascending to 30% B in 9.0 min, ascending to 90% B within 0.5 min and maintained for 4 min, and descending to 2% B within 0.5 min and maintained for 5 min. Fraction eluted at 7.0 to 7.5 min was collected, concentrated, and dried. ^1^H NMR and ^13^C NMR spectra of samples were performed at 600 and 150 MHz on Bruker AVIIIHD 600 NMR spectrometer (Bruker, Germany) respectively. The chemistry shifts were recorded in δ (ppm) and referenced to the solvent peaks (dimethyl sulfoxide (DMSO)-*d*_6_).

### Comparison of IMM-H004 Metabolism in Human and Rat Liver Microsomes

To compare the metabolic capability of IMM-H004 demethylation in rat and human liver microsomes, the kinetics of M1 and M2 formation in HLMs or RLMs were determined. Enzyme kinetic experiments were performed in triplicate. The incubation mixtures contained HLM/RLM (1 mg protein/ml), IMM-H004 (0.1–1,800 μM), and NADPH (1.2 mM) in a final volume of 0.2 ml Tris-HCl buffer (50 mM, pH 7.4) containing 5 mM MgCl_2_. The reaction was incubated at 37°C for 20 min and stopped by adding 200 μL of ice-cold acetonitrile containing IS (100 ng/ml). After vortex-mixing and centrifugation, M1 and M2 in the supernatant were analyzed by LC-MS/MS.

To explore glucuronidation difference of IMM-H004 between humans and rats, the kinetics of IMM-H004G formation in UDPGA-supplemented HLMs and RLMs were determined. The incubation mixtures consisted of HLMs or RLMs (0.025 mg protein/ml), alamethicin (50 μg/mg protein), IMM-H004 (0.5–2,500 μM), and UDPGA (5 mM) in a final volume of 0.2 ml Tris-HCl buffer (50 mM, pH 7.4) containing 5 mM MgCl_2_. HLM or RLM was preincubated with alamethicin on ice for 15 min. The reaction was incubated at 37°C for 10 min and quenched by adding 200 μL of ice-cold acetonitrile containing IS (100 ng/ml). After vortex-mixing and centrifugation, IMM-H004G in the supernatant was analyzed by LC-MS/MS.

### IMM-H004 Metabolism by Human Hepatocytes

Primary human hepatocytes were used to predict the metabolism of IMM-H004 in humans. Cryopreserved human hepatocytes were obtained from Bioreclamation IVT (Baltimore, MD, USA). Three different hepatocyte preparations (Lot CDP, ZHL, and DSX) were pooled in this study. Hepatocytes were resuspended and seeded in 96-well plates at a density of 0.7 × 10^6^ cells/ml. After 24 h of culture, cells were washed with Hanks’ balanced salt solution, followed by addition of 30 nM IMM-H004 dissolved in Hanks’ balanced salt solution. Incubations were carried out at 37°C up to 3 h under gentle shaking. Cell culture supernatant was collected within 3 h and determined by LC-MS/MS. All experiments were conducted in triplicate.

### Identification of Metabolizing Enzymes

To identify metabolizing enzymes, IMM-H004 was incubated with different types of human recombinant enzymes.

*CYP450s*. IMM-H004 was incubated in triplicate with 13 individual human complementary DNA (cDNA) expressed CYPs (CYP1A1, 1A2, 2A6, 2B6, 2C8, 2C9, 2C19, 2D6, 2E1, 2J2, 3A4, 4A11, 4F2, and 4F3) at 37°C. The incubation mixtures contained individual CYP450 enzymes (50 pmol/ml), IMM-H004 (1 μM), and NADPH regeneration system in a final volume of 0.2 ml Tris-HCl buffer (50 mM, pH 7.4) containing 5 mM MgCl_2_. The mixtures were incubated for 40 min before being quenched with two volumes of ice-cold acetonitrile containing IS (100 ng/ml). After vortex-mixing and centrifugation, M1 and M2 in the supernatant were analyzed by LC-MS/MS.

*UGTs*. IMM-H004 was incubated in triplicate with UGT1A1, 1A3, 1A4, 1A6, 1A7, 1A8, 1A9, 1A10, 12B4, 12B7, 12B10, 12B15, and 12B17 at 37°C. The incubation mixtures contained individual UGT enzyme (0.2 mg protein/ml), IMM-H004 (1 μM), and UDPGA (5 mM) in a final volume of 0.2 ml Tris-HCl buffer (50 mM, pH 7.4) containing 5 mM MgCl_2_. The mixtures were incubated for 30 min before being quenched with two volumes of ice-cold acetonitrile containing IS (100 ng/ml). After vortex-mixing and centrifugation, IMM-H004G in the supernatant was analyzed by LC-MS/MS.

*Sulfotransferases (SULTs)*. IMM-H004 was incubated in triplicate with SULT1A1, 1A1*1, 1A2, 1A3, 1C2, 1C4, 1E1, 1B1, and 2A1 at 37°C. The incubation mixtures contained individual SULT enzyme (0.1 mg protein/ml) and IMM-H004 (1 μM), PAPS (1 mM) in a final volume of 0.2 ml Tris-HCl buffer (50 mM, pH 7.4) containing 5 mM MgCl_2_. The mixtures were incubated for 60 min before being quenched with two volumes of ice-cold acetonitrile containing IS (100 ng/ml). After vortex-mixing and centrifugation, M4 in the supernatant was analyzed by LC-MS/MS.

### PK and PD Study in Rats

The plasma pharmacokinetics (PK) and pharmacodynamics (PD) of IMM-H004 and IMM-H004G were investigated in a cerebral ischemia-reperfusion rat model produced by middle cerebral artery occlusion/reperfusion (MCAO/R) after iv administration of IMM-H004. In brief, rats underwent a 1-h MCAO and then received IMM-H004 citrate (10 mg/kg, dissolved in saline to yield a concentration of 2 mg/ml) immediately after reperfusion, an effective dosing time as reported (Zuo et al., 2015; Yang et al., 2017). For sham surgery, all the arteries were exposed for the surgical period, but the filament was not inserted into the MCA. Blood samples were collected through external jugular vein cannula into heparinized tubes at 0.033, 0.167, 0.5, 1, 2, 4, 6, 8, 12, 24, 36, and 48 h postdose for IMM-H004 and IMM-H004G detection (50 μL of blood at each time point) and at 0.5, 1, 2, 4, 7, and 10 h postdose for malondialdehyde (MDA) detection (80 μL of blood at each time point). Plasma was immediately separated by centrifugation at 900×*g* for 5 min. Plasma concentrations of IMM-H004 and IMM-H004G in ischemia-reperfusion rats were determined by LC-MS/MS analysis. The PK parameters were calculated by noncompartmental analysis using WinNonlin Version 6.1 (Pharsight, Mountain View, CA). Plasma MDA concentrations in ischemia-reperfusion and sham rats within 24 h were estimated by enzyme-linked immunosorbent assay.

### Liquid Chromatography Tandem Mass Spectrometry Analysis

The LC-MS/MS system consisted of Shimadzu 30A UPLC and API 4000 triple quadruple mass spectrometer (AB SCIEX, USA). Data acquisition and analysis were accomplished using Analyst 1.5.2 software. The analytes and IS were chromatographed by injection of 1 μL sample. The mobile phases consisted of solvent A (0.5% formic acid in water) and solvent B (0.5% formic acid in methanol) at a flow rate of 0.3 ml/min on an Eclipse Plus C18 column (2.1 mm × 50 mm, 3.5 μm, Agilent, USA), and the operating temperature was 40°C. The elution condition was 10% solvent B for 0.7 min, ascending to 98% B in 2.3 min, holding for 3.0 min and reequilibrating to 10% B within 0.1 min and maintained for 2.4 min (Jiang JW, 2018). The specific transitions monitored were 305→248 for IMM-H004, 291→234 for M1, 291→248 for M2, 481→305 for IMM-H004G, 385→305 for M4, and 260→183 for IS.

### Neuroprotection of IMM-H004 and IMM-H004G

#### *In Vitro*

The neuroprotective activity of IMM-H004 and IMM-H004G was evaluated on PC12 cells damaged by oxygen-glucose deprivation (OGD). PC12 cells were purchased from the American Type Culture Collection. Cultures were maintained at 37°C in 5% CO_2_ in a humidified incubator. For cell viability assay, PC12 cells were incubated in 96-well plates at a density of 5 × 10^4^/ml for 24 h. In the sham group, PC12 cells were cultured in Dulbecco’s modified eagle medium supplemented with 5% fetal bovine serum and 5% equine serum (Gibco, USA). In the OGD group, PC12 cells were cultured in glucose-free Earle’s balanced salt solution supplemented with 15 mM Na_2_S_2_O_4_ for 2 h and then incubated with compounds of nerve growth factor (NGF, 10 μM), edaravone (10 μM, 50 μM), IMM-H004 (1 μM, 10 μM), and IMM-H004G (1 μM, 10 μM), respectively. After 24 h of incubation, MTT (5 mg/ml) was added into the cell cultures and incubated for an additional 4 h. Then the supernatant was removed and 100 μL DMSO was added. Absorbance was measured using an Ultramark microplate reader at a wavelength of 562 nm. The cell viability was expressed as a percentage of the absorbance density value of control cultures.

#### *In Vivo*

Transient MCAO/R was applied to compare the neuroprotection of IMM-H004 and IMM-H004G. Rats were fasted overnight with free access to water and randomly assigned to different groups. Rats were anesthetized with a mixture of 5% isoflurane and 95% oxygen and maintained with a mixture of 3% isoflurane and 97% oxygen during the surgical procedure. A 4-0 nylon thread, the tip of which was burned (diameter 0.36 mm), was inserted into the right internal carotid artery and advanced until the origin of the right MCA was occluded. After 60 min of the occlusion, the thread was withdrawn to allow reperfusion, and then the rats were returned to the chamber. Saline or edaravone at dose of 6 mg/kg or IMM-H004 citrate and IMM-H004G at dose of 10 mg/kg (dissolved in saline to yield a concentration of 2 mg/ml) were administered by intravenous injection immediately after reperfusion.

The rats were assessed for neurologic deficits at 24 h after reperfusion according to Zea Longa’s five-point scale. A score of 0 indicates no neurological deficit; a score of 1 indicates failure to extend left forepaw fully; a score of 2 indicates circling to the left; a score of 3 indicates falling to the left; a score of 4 indicates did not walk spontaneously and had a depressed level of consciousness; and a score of 5 indicates death. The animals without symptoms of neurological impairment or dying after the surgery were rejected. All animals were killed 24 h after reperfusion. Brains of the animals were removed and cut into 2 mm-thick slices, for a total of six slices per animal. The slices were immersed in a 2% solution of 2,3,5-triphenyltetrazolium chloride in phosphate buffered saline at 37°C for 20 min and then fixed in 4% formaldehyde overnight. Images of the slices were obtained with a scanner and a computer. The infarct area and the total area were calculated by tracing the areas on the computer screen with Image J. The percentage of the infarct volume was expressed as the infarct volumes (white parts)/the whole volume of the cortex.

### Statistical Analysis

Data are presented as mean ± SD or SEM. Statistical evaluation was performed using one-way analysis of variance. Significant difference was further performed in conjunction with the Student–Newman–Keuls method. Statistical significance was accepted at *p* < 0.05.

## Results

### Identification of IMM-H004 Metabolites *In Vivo* and *In Vitro*

**Figure 1:**
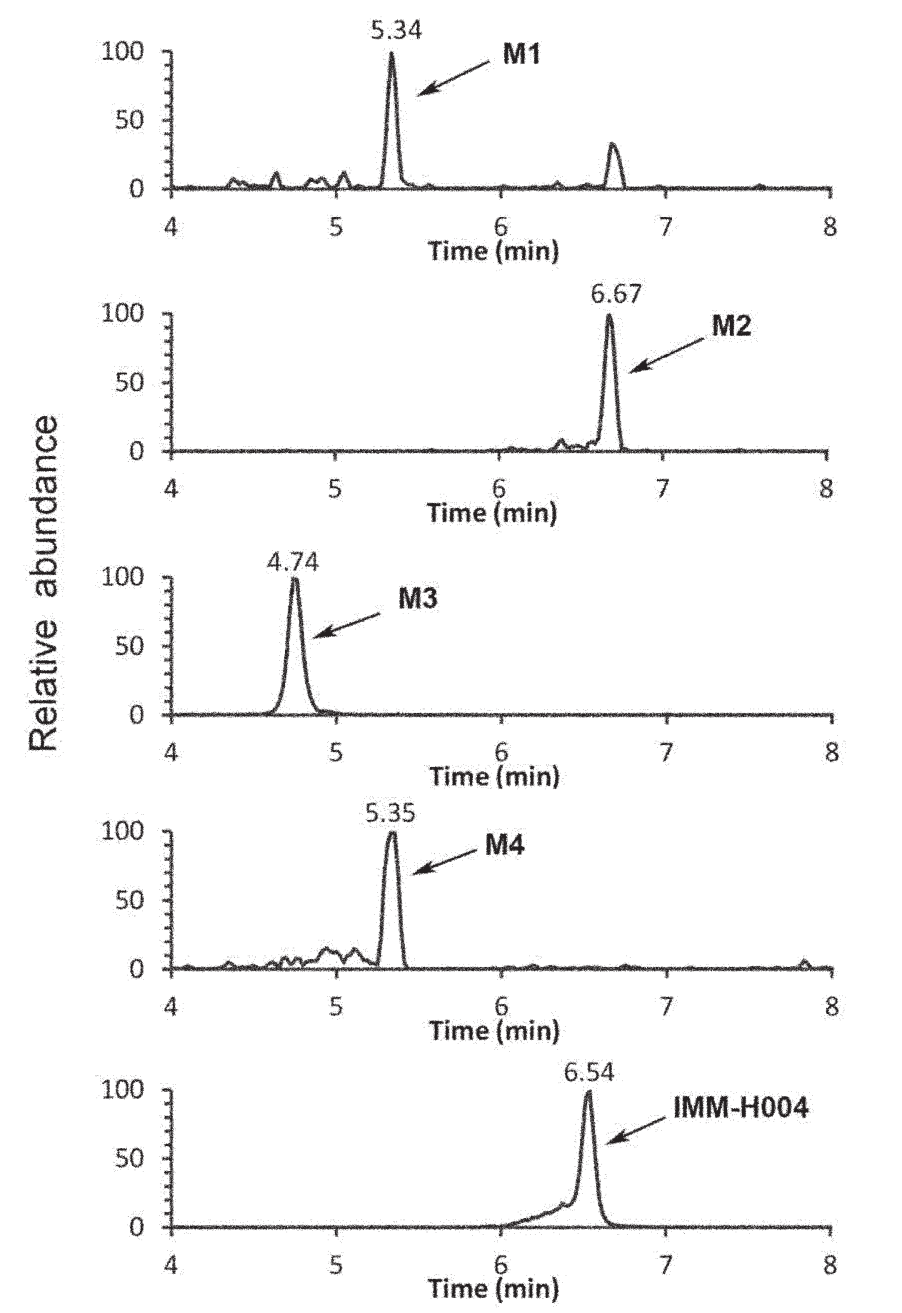
Representative extracted ion chromatogram of IMM-H004 and its metabolites in urine.

The protonated IMM-H004 molecule (*m/z* 305) was detected under positive ion mode, which further dissociated in MS^2^ to produce the fragment ions at *m/z* 248, *m/z* 220, *m/z* 192, and *m/z* 177 (**Table 1**). The metabolic profile of IMM-H004 in rat urine, feces, bile, and *in vitro* incubations was analyzed by LC-MS/MS. Four metabolites (M1, M2, IMM-H004G, and M4) of IMM-H004 were detected (**Figure 1**).

**Table 1:** Liquid chromatography–mass spectrometry–mass spectrometry (LC/MS^2^) data of IMM-H004 and its metabolites.

Metabolite	Retention time (min)	MS [M+H]^+^	*m/z* (relative abundance, %)
IMM-H004	6.54	305	248 (67), 220 (45), 192 (93), 177 (86)
M1	5.34	291	234 (100), 206 (34), 163 (65)
M2	6.67	291	248 (100), 220 (42), 192 (65), 177 (52)
IMM-H004G (M3)	4.74	481	305 (100)
M4	5.35	385	305 (100)

**Figure 2:**
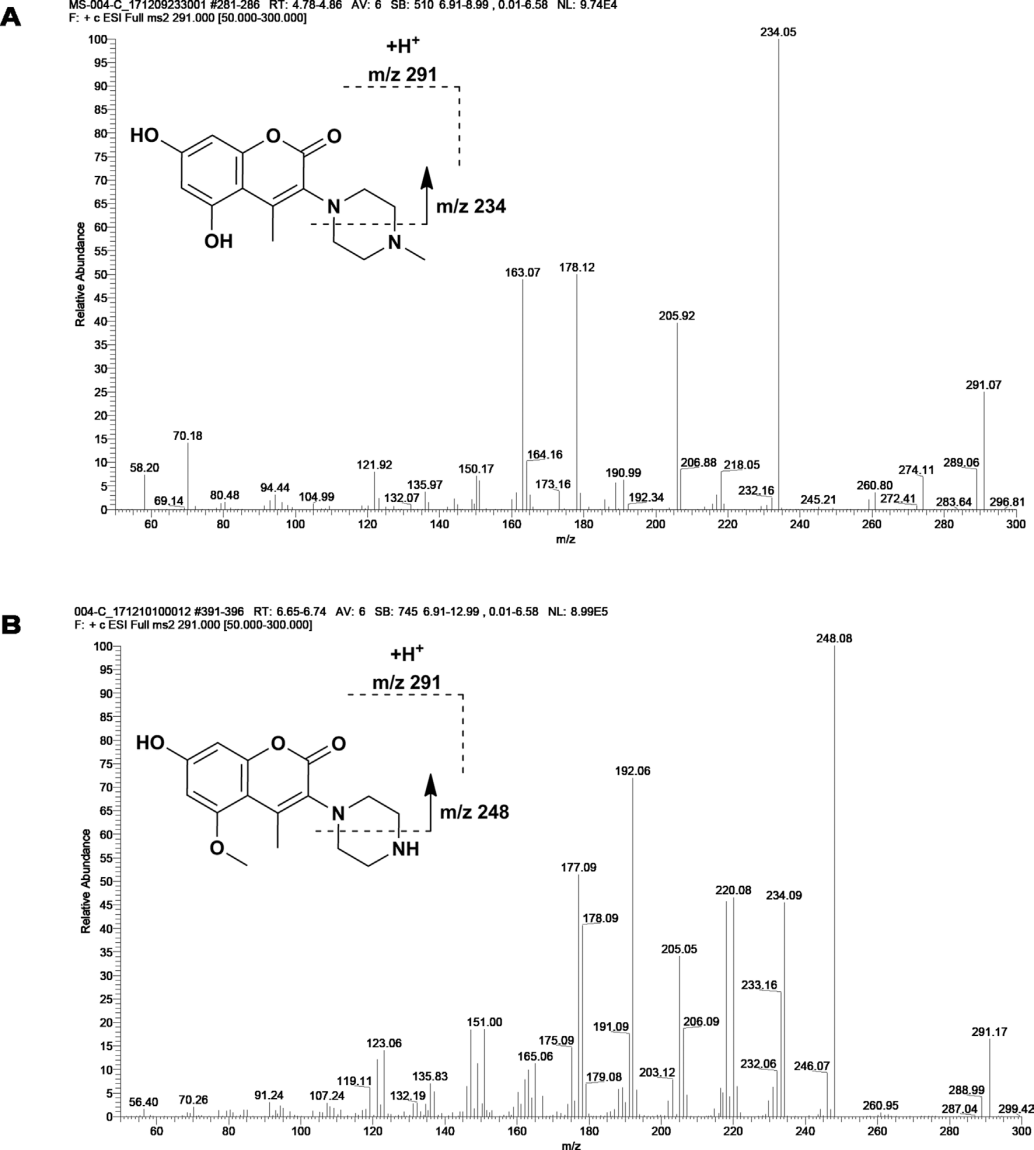
Fragmentation profiles of M1 **(A)** and M2 **(B)** on MS.

**Figure 3:**
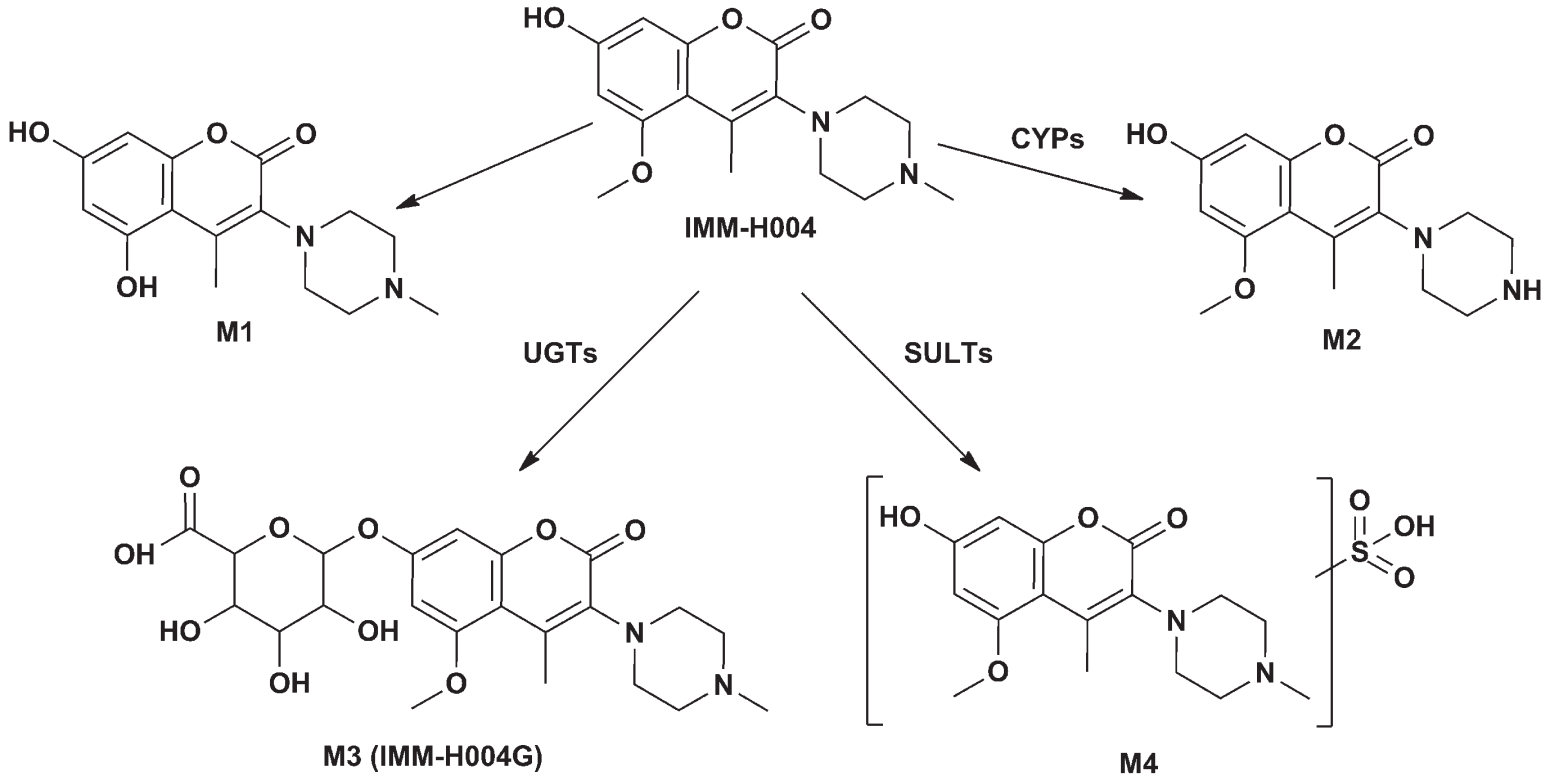
Possible metabolic pathway of IMM-H004.

M1 and M2 could be detected in rat urine, bile, feces, RLMs, and HLMs incubations. Both of them generated a protonated molecule of *m/z* 291 which was 14 Da smaller than that of the protonated parent compound, suggesting the loss of methyl. Upon collision-induced dissociation, the fragment ions *m/z* 234, *m/z* 206, and *m/z* 163 of M1 were 14 Da smaller than those of the parent drug, and the fragment ions 248, 220, 192, and 177 of M2 were consistent with those of the parent drug. Since the retention times and fragmentation profiles of M1 and M2 were consistent with those synthesized reference compounds, M1 and M2 were identified as 6-demethylation and N-demethylation IMM-H004, respectively (**Figures 2** and **3**).

M3 (named IMM-H004G) was present in rat urine, bile, RLMs, and HLMs incubations. It showed a protonated molecular ion at *m/z* 481, 176 Da higher than that of the parent drug, suggesting that it was a conjugate. The MS/MS spectrum of *m/z* 481 gave an intense ion at *m/z* 305, a loss of 176 Da from the precursor ion, indicating that it was a glucuronide conjugate. Treatment of urine with β-glucuronidase resulted in the disappearance of IMM-H004G. The structure of IMM-H004G was further characterized by NMR. ^13^C NMR data (in DMSO-*d*_6_) clearly showed the presence of a glucuronic acid moiety [δ_C_ 71.7 (C-4″), 72.9 (C-2″), 74.3 (C-5″), 76.3 (C-3″), 99.4 (C-1″), 171.5 (C-6″)]; the remaining NMR data were similar to those of IMM-004 (Sun MN, 2013). In the heteronuclear multiple bond correlation (HMBC) spectrum, the key HMBC correlations from H-1″ (δ_H_ 5.11, d, *J* = 7.2 Hz), H-6 (δ_H_ 6.57, d, *J* = 2.4 Hz), and H-8 (δ_H_ 6.64, d, *J* = 2.4 Hz) to C-7 (δ_C_ 159.3) were observed (**Table 2**). These results demonstrated that IMM-H004G was 7-*O*-β-glucuronide conjugate of IMM-H004 (**Figure 3**).

**Table 2:** NMR spectroscopic data for IMM-H004G (600 MHz, DMSO-*d*_6_)

Position	δ_C_	δ_H_ (*J* in Hz)
2	157.5	
3	130.3	
4	147.9	
4a	105.2	
5	158.8	
6	97.2	6.57 (d, *J* = 2.4)
7	159.3	
8	95.6	6.64 (d, *J* = 2.4)
8a	153.8	
1'	48.4	3.17 (2H, s)
2'	54.8	-^a^
1″	99.4	5.11 (d, *J* = 7.2)
2″	72.9	3.25 (m)
3″	76.3	3.32 (m)
4″	71.7	3.28 (m)
5″	74.3	3.79 (d, *J* = 9.6)
6″	171.5	
4-CH_3_	17.1	2.59 (s)
5-OCH_3_	56.3	3.86 (s)
N-CH_3_	45.2	2.33 (s)

a Not observed.

DMSO, dimethyl sulfoxide; NMR, nuclear magnetic resonance.

M4 was found in rat urine, bile, and human/rat liver cytosol incubations. It showed a protonated molecular ion at *m/z* 385, 80 Da higher than that of the parent drug, indicating the formation of a conjugate. The collisional activated decomposition product ion spectrum of *m/z* 385 showed an ion at *m/z* 305, a loss of 80 Da from the protonated molecular ion, suggesting the presence of a sulfate conjugate. Treatment of urine with sulfatase resulted in the disappearance of M4, further suggesting that M4 was a sulfated conjugate of IMM-H004.

Further quantitative analysis of rat urine, bile, and feces by LC-MS/MS showed that the total urinary IMM-H004G and IMM-H004 recovered over the 72-h sampling period was 72.5% of intake, and urinary IMM-H004G was 69.7% of intake. The recovery of IMM-H004G and IMM-H004 in bile accounted for 76.1% of intake, and the recovery of IMM-H004G accounted for 75.9% of intake. The fecal specimens mainly contained IMM-H004, accounting for 18% of the dose. Therefore, IMM-H004G was the main excretion form of the drug *in vivo*.

### IMM-H004 Metabolism in Liver Microsomes and Hepatocytes

**Figure 4:**
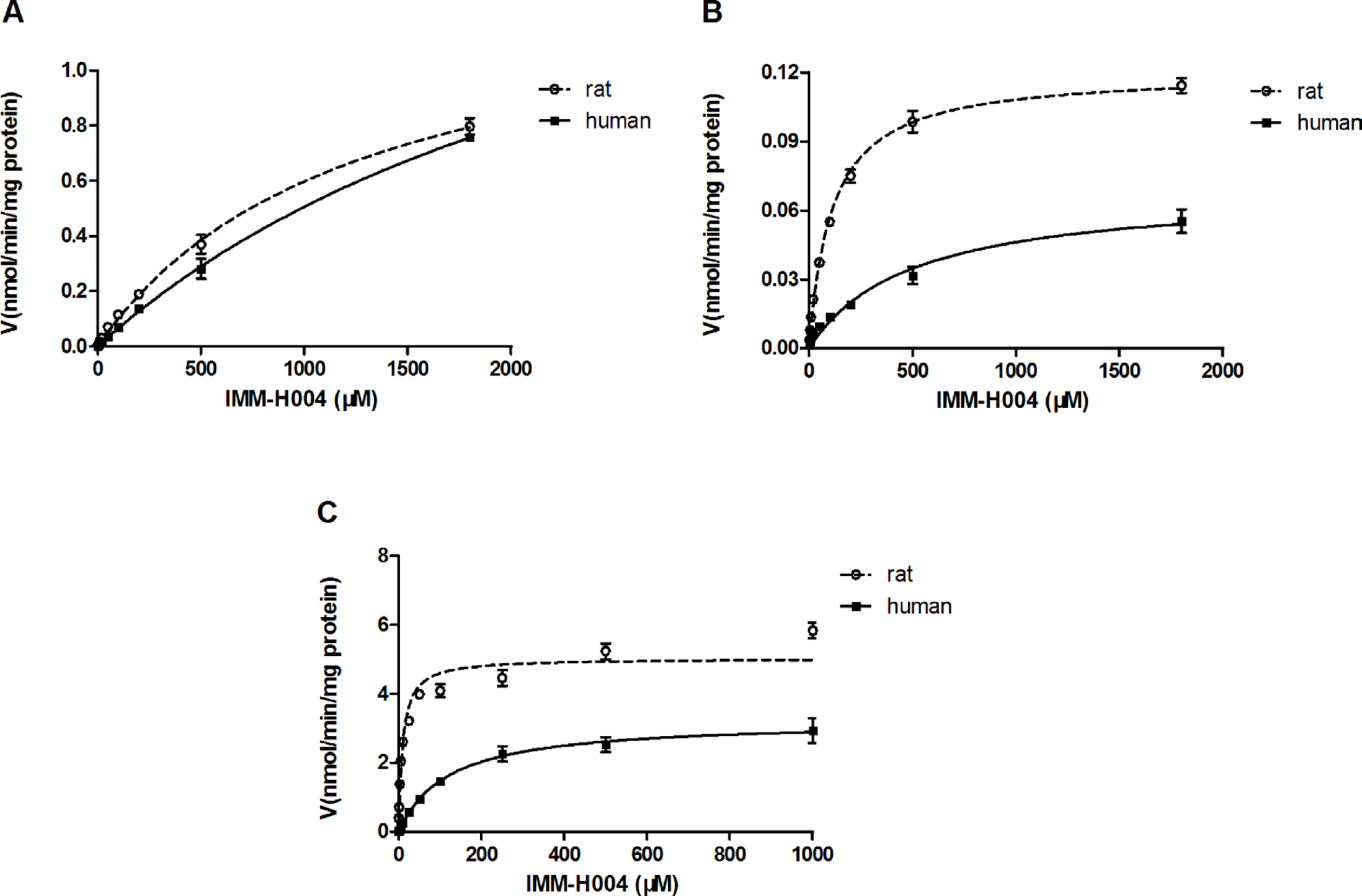
Michaelis–Menten kinetics of IMM-H004 demethylation and glucuronidation in rat and human liver microsomes. **(A)** Kinetic analysis of M1 generation by IMM-H004 demethylation; **(B)** kinetic analysis of M2 generation by IMM-H004 demethylation; **(C)** kinetic analysis of IMM-H004G generation by IMM-H004 glucuronidation. Error bars represent SD (*n* = 3).

Michaelis-Menten kinetic parameters clearly demonstrated the difference between demethylation and glucuronidation of IMM-H004 in RLM and HLM (**Figure 4**). The estimated apparent *K*_m_ and *V*_max_ values for demethylation and glucuronidation together with the intrinsic clearance are summarized in **Table 3**. Obviously, the maximal rate of N-demethylation (0.07–0.12 nmol/min/mg protein) was much lower than that of 6-demethylation (1.34–1.99 nmol/min/mg protein) and glucuronidation (3.25–5.04 nmol/min/mg protein) in both species. Besides, glucuronidation pathway exhibited at least 40-fold *V*_max_/*K*_m_ value compared with 6-demethylation. Therefore, glucuronidation was a high-capacity pathway. The glucuronidation *V*_max_ in rat samples (5.04 nmol/min/mg protein) was 1.6-fold higher than that in human samples (3.25 nmol/min/mg protein); this finding indicated that rats would have a greater capacity than humans for glucuronidation.

**Table 3:** Michaelis–Menten parameters for IMM-H004 demethylation and glucuronidation in rat and human liver microsomes.

Parameter	M1		M2		IMM-H004G	
	rat	human	rat	human	rat	human
*V*_max_ (nmol/min/mgprotein)^a^	1.34	1.99	0.12	0.07	5.04	3.25
*K*_m_ (μM)^a^	1,244.0	2,935.0	111.9	491.4	9.5	119.3
*CL*_int_ (μL/min/mg protein)^b^	1.08	0.68	1.08	0.14	529.83	27.20

a Nonlinear regression analysis was performed by software of GraphPad Prism to get Michaelis–Menten apparent parameters, and the analysis was carried out by the mean values of three concentrations at each time point.

b CL_int_was calculated by V_max_/K_m_.

**Figure 5:**
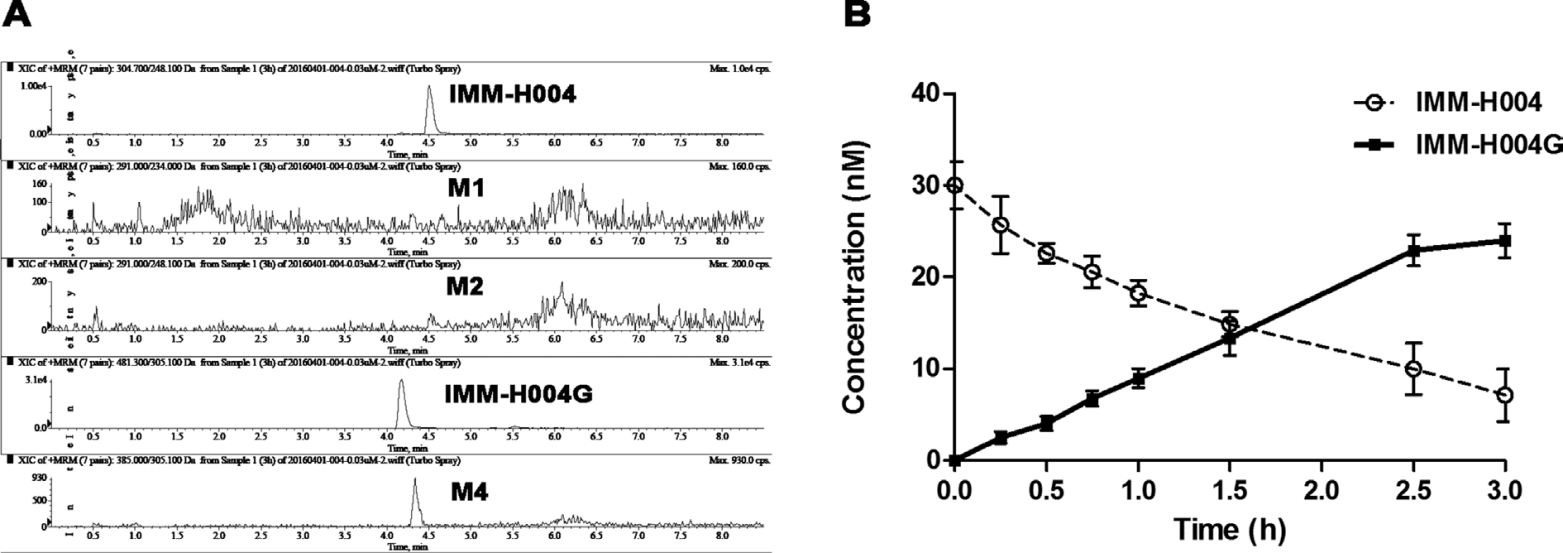
Metabolism of IMM-H004 by primary human hepatocytes. **(A)** Representative extracted ion chromatogram of IMM-H004 and its metabolites. The elution condition was 10% solvent B for 1 min, ascending to 98% B in 2 min, holding for 3.0 min, reequilibrating to 10% B within 0.1 min, and maintained for 2.4 min at a flow rate of 0.3 ml/min. **(B)** Production of IMM-H004G in human hepatocytes with 30 nM of IMM-H004. Error bars represent SD (*n* = 3).

IMM-H004G and trace of M4 could be detected in cell supernatant after IMM-H004 was incubated with hepatocytes, while M1 and M2 were not detectable (**Figure 5A**). After 3 h of human hepatocytes incubation, more than 70% of IMM-H004 was converted to IMM-H004G, and the total concentration of IMM-H004 and IMM-H004G was nearly equal to the initial concentration of IMM-H004 (30 nM) added in the cell supernatant (**Figure 5B**). The results indicated that IMM-H004G was the major metabolite of IMM-H004 in human hepatocytes, and the metabolic profile of IMM-H004 in humans was probably consistent with that of rats.

### Metabolism of IMM-H004 by cDNA-Expressed Human Metabolizing Enzymes

**Figure 6:**
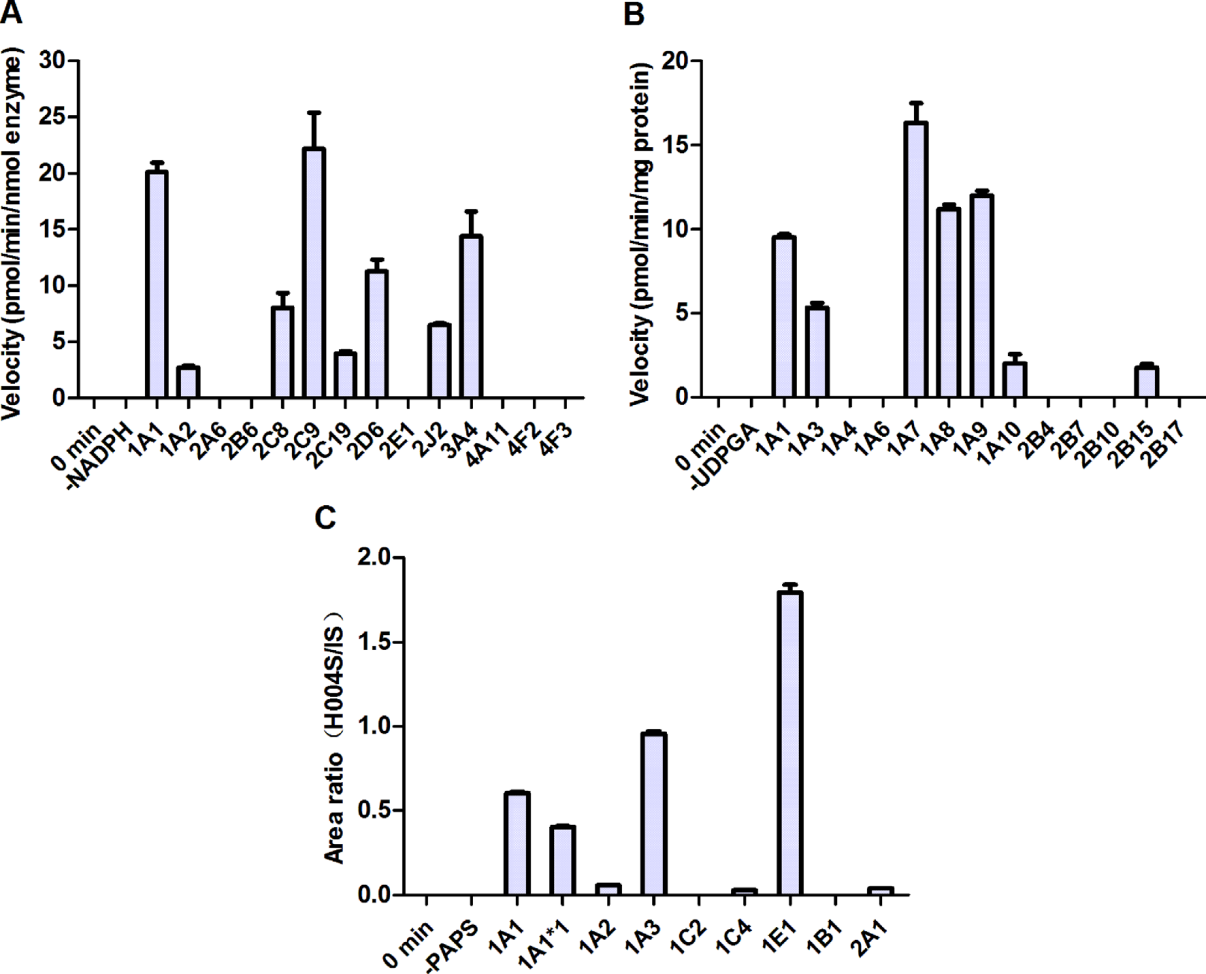
Metabolism of IMM-H004 by cDNA-expressed human metabolizing enzymes. **(A)** M2 formation by cytochromes P450 (CYP450s); **(B)** IMM-H004G formation by UDP-glucuronosyltransferases (UGTs); **(C)** M4 formation by sulfotransferases (SULTs). Error bars represent SD (*n* = 3).

To identify the enzymes involved in the formation of M1 and M2, a panel of cDNA-expressed recombinant CYP450 enzymes was screened for their activities. As shown in **Figure 6A**, conversion of IMM-H004 to M2 was catalyzed by CYP1A1, 2C9, 2D6, and 3A4, and to a lesser extent by CYP1A2, 2C8, 2C19, and 2J2. To make a better estimate of the relative contribution of each CYP450 enzyme to the overall clearance of IMM-H004 in humans, the enzyme activities were normalized by the average content of each enzyme in HLM (Gong et al., 2012). As a result, CYP2C9 and CYP3A4 were predicated to be the major contributors for the formation of M2. Meanwhile, M1 could not be detected in all CYP450 enzymes incubations, suggesting less possibility that these enzymes are involved in the formation of M1. So other phase I metabolizing enzymes responsible for M1 formation remain to be discovered.

To evaluate the activities of UGT enzymes for the formation of IMM-H004G, IMM-H004 was incubated with individual human cDNA-expressed UGT enzyme in the presence of UDPGA. As indicated in **Figure 6B**, conversion of IMM-H004 to IMM-H004G was catalyzed by UGT1A7, 1A9, 1A8, and 1A1 and to a lesser extent by UGT1A3, 1A10, and 2B15.

In addition, we examined the metabolism of IMM-H004 with individual human cDNA-expressed SULT enzyme in the presence of PAPS. The highest activity was observed with SULT1E1, followed by 1A3 and 1A1. Activities were markedly lower with SULT1A2, 1C4, and 2A1 and were negligible with SULT1C2 and 1B1 (**Figure 6C**).

### PK and PD Study of IMM-H004 in Rats

**Figure 7:**
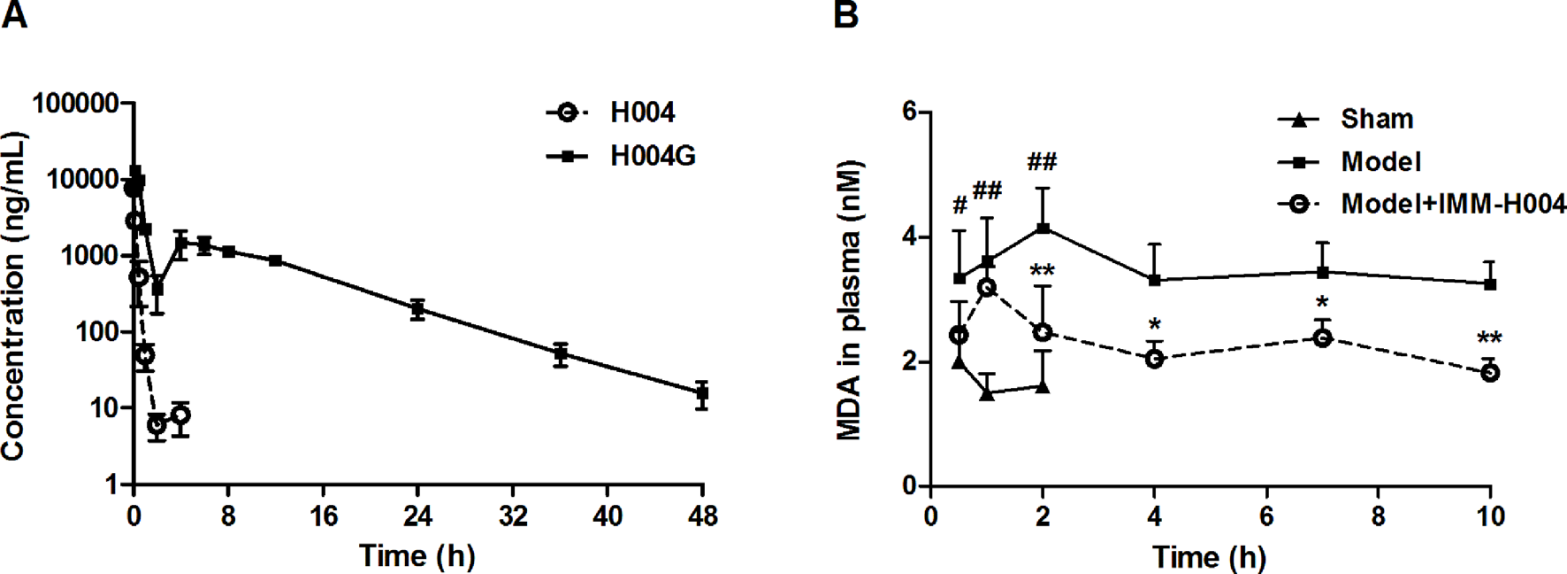
Plasma concentration-time profiles of IMM-H004 and IMM-H004G **(A)** and the effect on plasma malondialdehyde (MDA) suppression **(B)** in middle cerebral artery occlusion/reperfusion (MCAO/R) rats after IMM-H004 administration. IMM-H004 citrate (10 mg/kg dissolved in saline) was given immediately to MCAO rats after reperfusion *via* tail vein injection, and the collection of blood sample was timed according to the time of IMM-H004 iv injection. Error bars represent SD (*n* = 5). One-way analysis of variance was used, ^#^ *p* < 0.05, ^##^ *p* < 0.01 *vs*. Control, *p < 0.05, **p < 0.01 *vs*. Model group.

The mean plasma concentration-time profiles of IMM-H004 and IMM-H004G are presented in **Figure 7A**, and major PK parameters are shown in **Table 4**. After iv injection of IMM-H004 to MCAO/R rats, IMM-H004 eliminated rapidly with a short plasma elimination half-life (*t*_1/2β_) of 0.42 h. The concentration of IMM-H004G in plasma increased to a maximum of 13,020 ng/ml within 15 min after dosing, then declined slowly with a *t*_1/2β_ value of 6.61 h. The mean area under curve of IMM-H004G in plasma was 11.22-fold higher than that of IMM-H004. Meanwhile, no significant difference of PK was found between sham and MCAO/R rats (data not shown).

**Table 4:** PK parameters of IMM-H004 and IMM-H004G in MCAO/R rats after iv dosing of IMM-H004 citrate (10 mg/kg dissolved in saline).

Parameters	Unit	IMM-H004	IMM-H004G
*t*_1/2β_	h	0.42 ± 0.03	6.61 ± 0.86
*T*_max_	h	0.03 ± 0.00	0.17 ± 0.00
*C*_max_	ng/ml	7,463 ± 1,234	13,020 ± 923
MRT_(0-_ *_t_*_)_	h	0.18 ± 0.01	7.89 ± 0.57
MRT_(0-∞)_	h	0.19 ± 0.02	8.16 ± 0.65
AUC_(0-_ *_t_*_)_	h×ng/ml	1,638 ± 245	28,948 ± 2,017
AUC_(0-∞)_	h×ng/ml	1,643 ± 247	29,103 ± 2,049

Data are expressed as mean ± SD (n = 5).

AUC, area under the plasma concentration–time curve; MRT, mean residence time.

The effect of IMM-H004 on MDA levels is shown in **Figure 7B**. After 0.5 h of reperfusion, the plasma MDA level in MCAO/R rats was significantly higher than that in the sham group. IMM-H004 was shown to significantly lower the increased MDA level at 2 h and maintained for at least 10 h after administration compared to the MCAO/R group.

So the PK-PD data indicated that, compared with the parent drug, IMM-H004G exhibited a longer exposure time and higher exposure level and therefore had a better correlation with the duration of drug efficacy.

### Neuroprotection of IMM-H004 and IMM-H004G in Oxygen-Glucose Deprivation-Injured PC12 Cells

**Figure 8:**
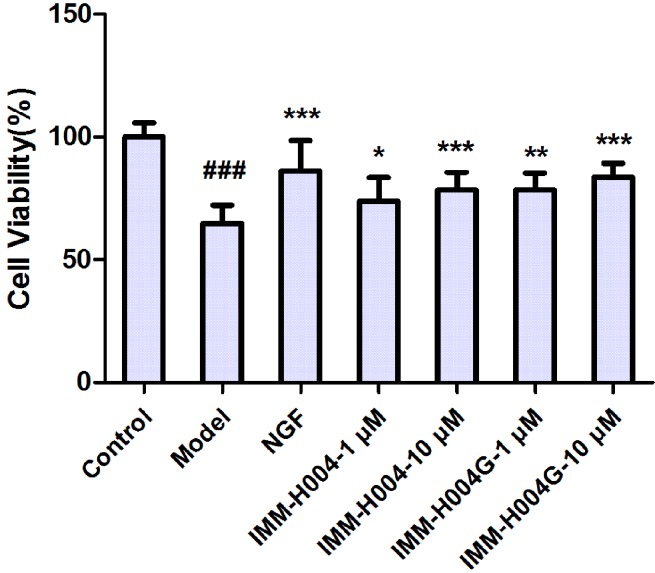
Neuroprotective activity of IMM-H004 and IMM-H004G against oxygen-glucose deprivation (OGD)-induced neuronal injury in PC12 cells. Error bars represent SD (*n* = 12). One-way analysis of variance was used, ^###^ *p* < 0.001 *vs*. Control, *p < 0.05, **p < 0.01, ***p < 0.001 *vs*. Model group.

The neuroprotective activity against OGD-induced neuronal injury in PC12 cells by IMM-H004 and IMM-H004G at 1 and 10 μM were evaluated by cell viability assay. As shown in **Figure 8**, cell viability of the model group decreased to 64.9% of the control group (*p* < 0.001). Cells treated with IMM-H004 and IMM-H004G at concentrations of 1 and 10 μM had significant (*p* < 0.05) preservation of viability. In addition, only trace of IMM-H004 could be detected in PC12 cultures after 24 h of incubation with IMM-H004G, much lower than the effective concentration of 1 μM *in vitro*. The result supports the neuroprotective activity of IMM-H004G.

**Figure 9:**
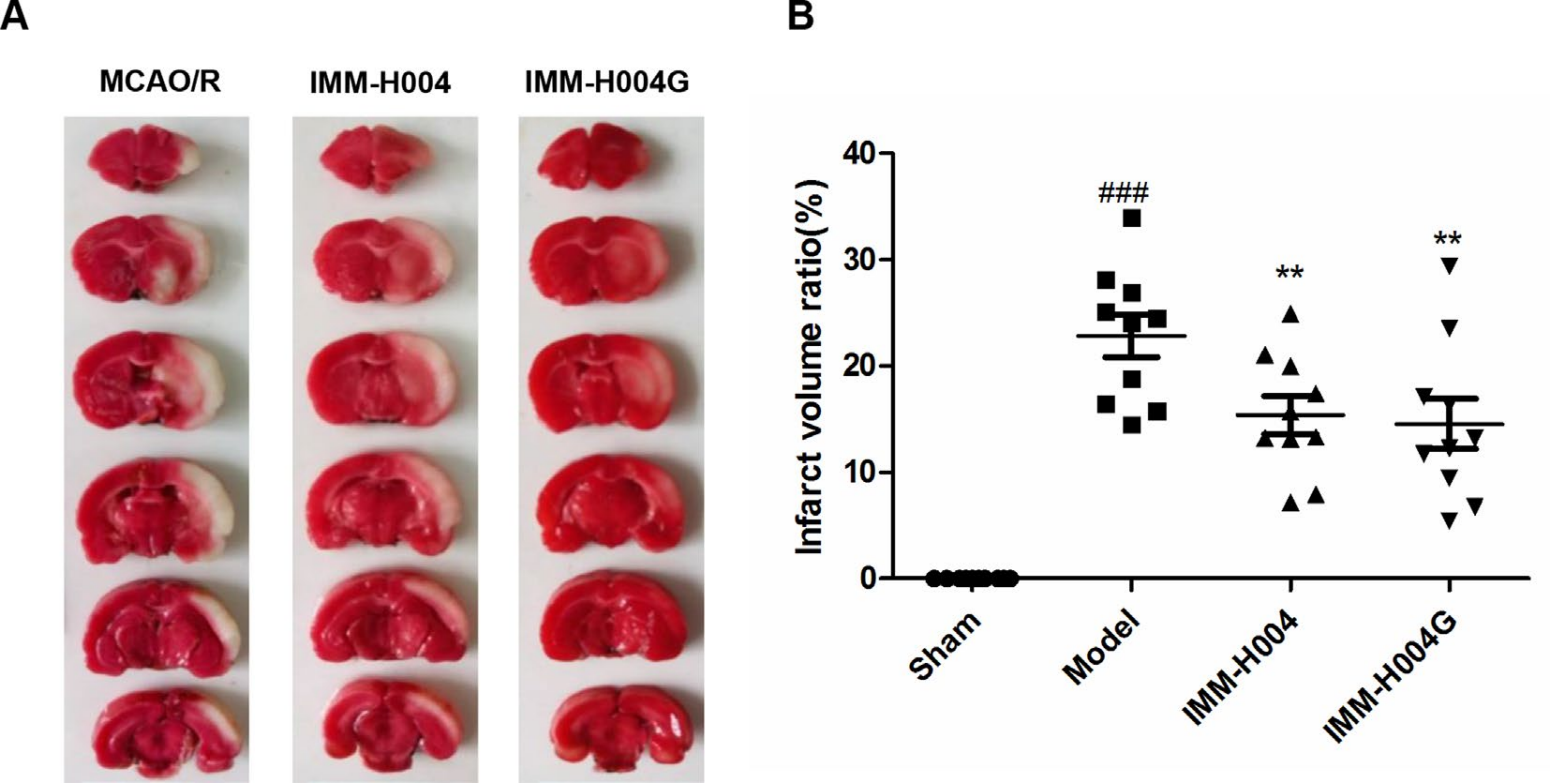
Effect of IMM-H004 and IMM-H004G on infarct volume of MCAO/R rats. **(A)** Representative brain slices stained by 2,3,5-triphenyltetrazolium chloride; **(B)** quantitative evaluation of infarct volume. Error bars represent SEM (*n* = 10). One-way analysis of variance was used, ^###^ *p* < 0.001 *vs*. Sham, **p < 0.01 *vs*. Model group.

**Figure 10:**
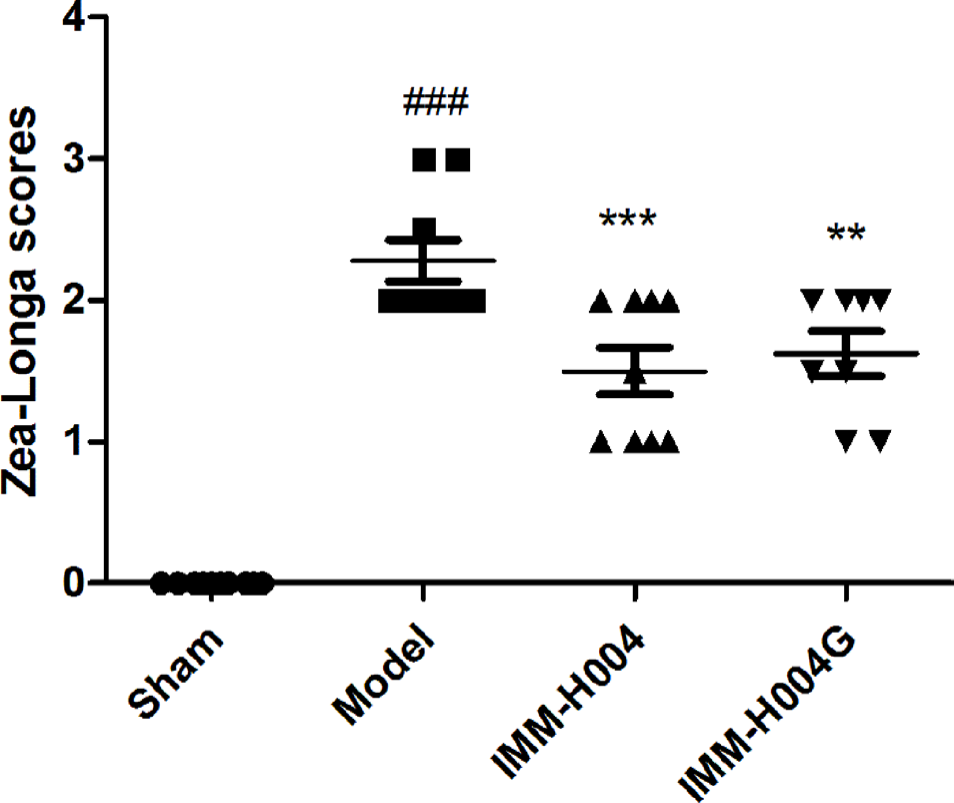
Effect of IMM-H004 and IMM-H004G on neurological behavior of MCAO/R rats. Error bars represent SEM (*n* = 10). One-way analysis of variance was used, ^###^ *p* < 0.001 *vs*. Sham, **p < 0.01, ***p < 0.001 *vs*. Model group.

Likewise, IMM-H004 and IMM-H004G significantly decreased brain infarct volume and neurological deficits following MCAO/R. At 24 h after ischemia-reperfusion, model rats exhibited visible intracerebral damage (infarct volume, 22.8%) and major neurological deficits. In rats treated with IMM-H004 and IMM-H004G, the infarct volume was markedly reduced (*p* < 0.01) (**Figure 9**). It was accompanied by a significant improvement (*p* < 0.01) of the neurological test score (**Figure 10**).

## Discussion

Drug metabolism research is an essential part of drug discovery and development. A comprehensive understanding of metabolic pathways and analysis of the concentration-effect relationship of new candidates and major metabolites can help us to reveal the pharmacoactive substances of a drug and provide useful information for drug development and clinical application.

IMM-H004 has been shown to have significant neuroprotective effect on a variety of pathological models. It could block chemokine-like factor 1-C27-induced calcium mobilization and chemotaxis, decrease the toxicity of Aβ, reduce inflammatory response, and improve blood–brain barrier function (Li et al., 2010; Song et al., 2013; Ji et al., 2014; Song et al., 2014; Zuo et al., 2014; Zuo et al., 2015; Chu et al., 2017; Niu et al., 2017). Unexpectedly, IMM-H004 underwent extremely short elimination half-life (0.19 h) in rat plasma and brain (Zhang et al., 2017). However, due to the low exposure and short elimination half-life of IMM-H004, it is difficult to explain its pharmacology activity. As such, it is very meaningful to investigate how the effect of IMM-H004 lasts for a long time and whether pharmacologically active metabolites are present. Subsequently, the metabolism of IMM-H004 was systematically investigated *in vitro* (with HLMs, human hepatocytes, and RLMs) and in rats *in vivo*.

Four metabolites of IMM-H004 including demethylated metabolites M1 and M2, glucuronide conjugate IMM-H004G, and sulfated conjugate M4 were identified *in vitro*. Enzyme kinetics analysis demonstrated that demethylation of IMM-H004 was a low capacity and affinity pathway. In contrast to demethylation, glucuronidation offered high affinity and capacity in both RLM and HLM. These results indicate that glucuronidation may play an important role in the metabolism of IMM-H004. The major metabolites of IMM-H004 observed *in vivo* in rats were consistent with that *in vitro*. After iv administration of IMM-H004, most of the drug was recovered in bile and urine as IMM-H004G, indicating that IMM-H004G was the major metabolite of the drug in rats.

Many functional groups can react with UDPGA to form *O*-, *N*- and *S*-glucuronides, respectively (Bock, 2015). IMM-H004 contains a hydroxyl group and a tertiary amine on the piperazine ring in its structure. Therefore, there are two possibilities for glucuronide metabolites of IMM-H004. Actually, we detected only one glucuronide metabolite of IMM-H004 by LC-MS/MS. Our NMR results confirmed that the glucuronide metabolite was the phenolic glucuronide. Like other compounds, the hydroxyl group appears to be a more active group that forms glucuronide.

It is well known that conjugation of hydroxyl groups of phenols can occur with both glucuronate and sulfate and that there are species differences in glucuronidation or sulfation rates between animals and humans due to different expression levels of UGT and SULT subtypes (Vaidyanathan and Walle, 2002). Due to the lack of M4 reference standards, the kinetics of sulfated conjugate of IMM-H004 in liver microsomes cannot be compared with that of glucuronide conjugates, so the metabolite profile of IMM-H004 was further investigated in primary human hepatocyte to predict the metabolism of IMM-H004 in humans. The results showed that although a small amount of sulfated conjugate can be detected, IMM-H004 was predominantly metabolized to IMM-H004G in human hepatocytes. Therefore, glucuronidation may be the primary metabolic pathway of IMM-H004 in humans, similar to coumarin, a precursor compound of IMM-H004 (Ford et al., 2001; Wang et al., 2006; Leonart et al., 2017). But considering the glucuronidation clearance in RLM is much higher than that of HLM, the role of glucuronide conjugate in the metabolism and disposition of IMM-H004 in humans needs to be further investigated *in vivo*.

Glucuronidation is known to be catalyzed by a family of UGT enzymes. Human UGTs, consisting of at least 22 proteins, are divided into five subfamilies including UGT1A, 2A, 2B, 3A, and 8A on the basis of sequence identity. Members of the UGT1A and 2B subfamilies play a key role in drug metabolism (Rowland et al., 2013). Since the induction/inhibition and genetic polymorphism of UGTs are associated with drug therapy strategy and safety, it is important to identify the major metabolizing enzymes that act on a drug to predict interindividual variability in drug exposure and the potential of drug–drug interactions.

To determine the UGTs involved in IMM-H004 glucuronidation, recombinant human UGTs were applied. Our results indicated that UGT1A7, 1A9, 1A8, and 1A1 were the most active enzymes toward the glucuronidation of IMM-H004. Other UGTs also catalyzed the reaction, albeit less efficiently. Therefore, multiple UGT isoforms are involved in the IMM-H004 glucuronidation. When a drug is metabolized by a single enzyme, changing the enzyme activity is more likely to have a marked effect on the overall PK of the compound. Coadministration of drugs results in an increased likelihood of drug interactions. In contrast, the involvement of multiple UGT enzymes in the metabolism of IMM-H004 correspondingly makes it less likely that the PK profile of IMM-H004 was affected by other drugs. Since the abundant isoforms of UGT enzyme expression in human liver are UGT2B7, 1A4, 2B4, 1A1, 2B15, and 1A9, followed by UGT1A6 and 1A3, while UGT1A7 and 1A8 are mainly expressed in extrahepatic tissues (Izukawa et al., 2009; Achour et al., 2017; Tourancheau et al., 2018), it is anticipated that UGT1A1 and 1A9 are the major contributors for the formation of IMM-H004G in humans. It has been reported that hydroxycoumarin derivatives were good substrates of UGT. For example, 4-methylumbelliferone has been widely used as a nonspecific probe substrate for the evaluation of recombinant human UGTs activity (Uchaipichat et al., 2004). Although a variety of UGTs participate in the glucuronidation of hydroxycoumarins (Dong et al., 2012; Shan et al., 2014), UGT1A9 with greater capacity in bulk and complex phenol glucuronidation generally exhibits the highest catalytic activity (Ethell et al., 2002; Liang et al., 2010; Xia et al., 2014). Our data are consistent with these literatures.

PK/PD studies can reveal the relationship between drug concentration and efficacy, which helps to understand the mechanism of drug action. MCAO/R rat models were applied for IMM-H004 PK-PD study. MDA, the earliest indicator to reflect the efficacy of IMM-H004, served as a biomarker in plasma for PD, which was also because correlation between plasma MDA levels and severity of the disease was reported on both acute ischemic stroke patients and MCAO/R rats (Awooda et al., 2015; Jena et al., 2017). In line with previous reports, plasma MDA levels of our experiment were significantly increased in MCAO/R rats. IMM-H004 treatment was able to reduce MDA levels in MCAO/R rats, and the beneficial effect persisted for at least 10 h after treatment. As IMM-H004 is eliminated rapidly with a short plasma elimination half-life (0.42 h at 10 mg/kg, longer than *t*_1/2β _0.19 h at 6 mg/kg reported before, indicating that the drug elimination may be saturated at 10 mg/kg and there may be differences between experiments in different batches of animals), the exposure of IMM-H004 cannot adequately explain the duration of PD effect. IMM-H004G has a longer half-life and greater exposure in blood circulation than IMM-H004. Our previous brain microdialysis study also indicated that the exposure of IMM-H004G in rat brain extracellular fluid was 10.5-fold higher than that of IMM-H004 (Jiang et al., 2018). Therefore, IMM-H004G is the predominant form present in the body and drug target tissue. As the PK profile of IMM-H004G was consistent with the MDA inhibition curve, we speculated that IMM-H004G was likely to be an active metabolite.

Glucuronidation is generally considered as a process of detoxification and inactivation, because glucuronides usually possess less intrinsic biological or chemical activity than their parent forms and exhibit higher polarity and excretability. But there are exceptions; some glucuronide conjugates are active and contribute to pharmacological activities. The most typical examples with in-depth research were morphine-6-*O*-glucuronide and quercetin-3-*O*-glucuronide: Analgesic effect of morphine-6-*O*-glucuronide was achieved *via* activation of mu-opioid receptors, a G-protein-coupled receptor (Frölich et al., 2011). In addition, morphine-6-*O*-glucuronide was predominantly trapped in the extracellular fluid of brain with a high AUC value and therefore durably available to bind at opioid receptors inducing more potent central analgesia than morphine (Stain-Texier et al., 1999). Quercetin-3-*O*-glucuronide was also reported as an active compound which could inhibit intracellular reactive oxygen species in mouse fibroblast 3T3 cells induced by H_2_O_2_ attack and suppress invasion of MDA-MB-231 breast cancer cells and matrix metalloproteinase 9 (MMP-9) induction (Shirai et al., 2002; Yamazaki et al., 2014). Although hydroxycoumarin derivatives exhibit various biological activities and are susceptible to glucuronidation, there is no report on the biological activity of coumarin glucuronide conjugates. We investigated the neuroprotective effect of IMM-H004G.

IMM-H004G exhibited similar neuroprotection to that of parent compound *in vitro* and *in vivo*, which provided evidence that IMM-H004G may play a role in the neuroprotection of IMM-H004. Previous studies showed that IMM-H004 with high lipophilicity and low molecular size could easily cross the blood–brain barrier (*T*_max_ = 0.21 h) (Jiang et al., 2018). Therefore, we speculated that IMM-H004 may perform its activity rapidly, whereas IMM-H004G, with slower elimination and greater exposure in plasma and brain (Jiang et al., 2018), may contribute to the maintenance of anticerebral ischemia efficacy of IMM-H004 at least partly.

In addition, the secondary peak of IMM-H004G in **Figure 7**, more than 70% recovery of the drug in both biliary and urine mainly as IMM-H004G and undetectable IMM-H004G in feces, suggested together the existence of drug enterohepatic circulation. The enterohepatic circulation ensured reabsorption and persistence of the drug, and glucuronidation was the basis of enterohepatic circulation process. As a result, IMM-H004G may also contribute to anti-cerebral ischemia efficacy indirectly by improving the PK behavior of the drug.

Meanwhile, it was worth noting that the metabolite M1 also showed neuroprotective activity in cell cultures. Despite low exposure *in vivo*, M1 had an IC_50_ value of 2.12 × 10^−8^ M as an antagonist of the potent stroke target chemokine-like factor 1 in previous research (Li et al., 2010; Kong et al., 2011; Sun et al., 2013; Kong et al., 2014). And we cannot rule out the existence possibility of other unknown active metabolites, the lagged effect of IMM-H004 by the time required from cell signaling to biological effects, and the generation of IMM-H004 from IMM-H004G in certain microenvironment of target tissue. So the duration effect of IMM-H004 may be the result of a collective effect of IMM-H004, IMM-H004G, and other active metabolites. Wherefore, more comprehensive and in-depth research needs to be done in the future.

## Conclusion

In conclusion, four metabolites of IMM-H004 including demethylated metabolites, glucuronide conjugate IMM-H004G, and sulfated conjugate were detected *in vitro* and *in vivo*. Multiple drug metabolizing enzymes, including CYPs, UGTs, and SULTs, are involved in IMM-H004 metabolism. IMM-H004G is the major metabolite of IMM-H004 in rats and in human hepatocyte. The exposure and duration of IMM-H004G in MCAO/R rats are greater than that of IMM-H004. Notably, IMM-H004G exhibits a similar neuroprotective activity to that of the parent drug both *in vitro* and *in vivo*. IMM-H004G, at least in part, contributes to the maintenance of anticerebral ischemia efficacy of IMM-H004.

## Data Availability Statement

All datasets generated for this study are included in the manuscript and/or the supplementary files.

## Ethics Statement

This study was carried out in accordance with the recommendations of Guide for the use and care of laboratory animals, the Institute Animal Care and Welfare Committee. The protocol was approved by the Animal Care and Welfare Committee of Peking Union Medical College.

## Author Contributions

ZZ, DL, JJ, and XZ carried out the experiments; KX and JD synthesized IMM-H004G; XS and SC analyzed the experimental results; ZZ, DL, and LS wrote the manuscript; LS, NC, and YL designed the experiments and provided funds.

## Funding

This work was supported by the National Natural Science Foundation of China (81603190, 81603315, and 81803810), Drug Innovation Major Project (2018ZX09711001-002-001), CAMS Innovation Fund for Medical Sciences (2016-I2M-3-011), and the Fundamental Research Funds for the Central Universities (3332018091).

## Conflict of Interest Statement

The authors declare that the research was conducted in the absence of any commercial or financial relationships that could be construed as a potential conflict of interest.

## References

[B1] AchourB.DantonioA.NiosiM.NovakJ. J.FallonJ. K.BarberJ. (2017). Quantitative characterization of major hepatic UDP-glucuronosyltransferase enzymes in human liver microsomes: comparison of two proteomic methods and correlation with catalytic activity. Drug Metab. Dispos. 45, 1102–1112. 10.1124/dmd.117.076703 28768682

[B2] Awooda H. A. Lutfi M. F. Sharara G. G. M. Saeed A. M. (2015). Oxidative/nitrosative stress in rats subjected to focal cerebral ischemia/reperfusion. Int. J. Health Sci. (Qassim) 9, 17–24. 10.12816/0024679 25901129 PMC4394935

[B3] Bock K. W. (2015). Roles of human UDP-glucuronosyltransferases in clearance and homeostasis of endogenous substrates, and functional implications. Biochem. Pharmacol. 96, 77–82. 10.1016/j.bcp.2015.04.020 25937523

[B4] Chu S. F. Zhang Z. Zhang W. Zhang M. J. Gao Y. Han N. (2017). Upregulating the expression of survivin-HBXIP complex contributes to the protective role of IMM-H004 in transient global cerebral ischemia/reperfusion. Mol. Neurobiol. 54, 524–540. 10.1007/s12035-015-9673-5 26742528

[B5] Dong R. H. Fang Z. Z. Zhu L. L. Liang S. C. Ge G. B. Yang L. (2012). Investigation of UDP-glucuronosyl transferases (UGTs) inhibitory properties of carvacrol. Phytother. Res. 26, 86–90. 10.1002/ptr.3525 21544887

[B6] Ethell B. T. Ekins S. Wang J. Burchell B. (2002). Quantitative structure activity relationships for the glucuronidation of simple phenols by expressed human UGT1A6 and UGT1A9. Drug Metab. Dispos. 30, 734–738. 10.1124/dmd.30.6.734 12019203

[B7] Ford R. A. Hawkins D. R. Mayo B. C. Api A. M. (2001). The in vivo dermal absorption and metabolism of [4-14C] coumarin by rats and by human volunteers under simulated conditions of use in fragrances. Food Chem. Toxicol. 39, 153–162. 10.1016/S0278-6915(00)00123-X 11267709

[B8] Frölich N. Dees C. Paetz C. Ren X. Lohse M. J. Nikolaev V. O. (2011). Distinct pharmacological properties of morphine metabolites at G(i)-protein and β-arrestin signaling pathways activated by the human μ-opioid receptor. Biochem. Pharmacol. 81, 1248–1254. 10.1016/j.bcp.2011.03.001 21396918

[B9] Gong J. Gan J. Iyer R. A. (2012). Identification of the oxidative and conjugative enzymes involved in the biotransformation of brivanib. Drug Metab. Dispos. 40, 219–226. 10.1124/dmd.111.042457 21989950

[B10] Izukawa T. Nakajima M. Fujiwara R. Yamanaka H. Fukami T. Takamiya M. (2009). Quantitative analysis of UDP-glucuronosyltransferase (UGT) 1A and UGT2B expression levels in human livers. Drug Metab. Dispos. 37, 1759–1768. 10.1124/dmd.109.027227 19439486

[B11] Jena I. Nayak S. R. Behera S. Singh B. Ray S. Jena D. (2017). Evaluation of ischemia-modified albumin, oxidative stress, and antioxidant status in acute ischemic stroke patients. J. Nat. Sci. Biol. Med. 8, 110–113. 10.4103/0976-9668.198346 28250685 PMC5320811

[B12] Jiang J. Zhang Z. Zou X. Wang R. Bai J. Zhao S. (2018). Determination of IMM-H004 and its active glucuronide metabolite in rat plasma and Ringer’s solution by ultra-performance liquid chromatography-tandem mass spectrometry. J. Chromatogr. B Analyt. Technol. Biomed. Life Sci. 1074–1075, 16–24. 10.1016/j.jchromb.2017.12.030 29329091

[B13] Ji H. J. Wang D. M. Hu J. F. Sun M. N. Li G. Li Z. P. (2014). IMM-H004, a novel courmarin derivative, protects against oxygen-and glucose-deprivation/restoration-induced apoptosis in PC12 cells. Eur. J. Pharmacol. 723, 259–266. 10.1016/j.ejphar.2013.11.023 24291097

[B14] Kong L. L. Hu J. F. Zhang W. Yuan Y. H. Ma K. L. Han N. (2011). Expression of chemokine-like factor 1 after focal cerebral ischemia in the rat. Neurosci. Lett. 505, 14–18. 10.1016/j.neulet.2011.09.031 21964493

[B15] Kong L. L. Wang Z. Y. Han N. Zhuang X. M. Wang Z. Z. Li H. (2014). Neutralization of chemokine-like factor 1, a novel C-C chemokine, protects against focal cerebral ischemia by inhibiting neutrophil infiltration via MAPK pathways in rats. J. Neuroinflammation 11, 112. 10.1186/1742-2094-11-112 24946684 PMC4080607

[B16] Lee X. R. Xiang G. L. (2018). Effects of edaravone, the free radical scavenger, on outcomes in acute cerebral infarction patients treated with ultra-early thrombolysis of recombinant tissue plasminogen activator. Clin. Neurol. Neurosurg. 167, 157–161. 10.1016/j.clineuro.2018.02.026 29501045

[B17] Leonart L. P. Gasparetto J. C. Pontes F. L. D. Cerqueira L. B. de Francisco T. M. G. Pontarolo R. (2017). New metabolites of coumarin detected in human urine using ultra performance liquid chromatography/quadrupole-time-of-flight tandem mass spectrometry. Molecules 22, 2031. 10.3390/molecules22112031 PMC6150206 29165357

[B18] Liang S. C. Ge G. B. Liu H. X. Zhang Y. Y. Wang L. M. Zhang J. W. (2010). Identification and characterization of human UDP-glucuronosyltransferases responsible for the in vitro glucuronidation of daphnetin. Drug Metab. Dispos. 38, 973–980. 10.1124/dmd.109.030734 20176691

[B19] Li G. Wang D. Sun M. Li G. Hu J. Zhang Y. (2010). Discovery and optimization of novel 3-piperazinyl coumarin antagonist of chemokine-like factor 1 with oral antiasthma activity in mice. J. Med. Chem. 53, 1741–1754. 10.1021/jm901652p 20099827

[B20] Meschia J. F. Bushnell C. Boden-Albala B. Braun L. T. Bravata D. M. Chaturvedi S. (2014). Guidelines for the primary prevention of stroke: a statement for healthcare professionals from the American Heart Association/American Stroke Association. Stroke 45, 3754–3832. 10.1161/STR.0000000000000046 25355838 PMC5020564

[B21] Neuhaus A. A. Couch Y. Hadley G. Buchan A. M. (2017). Neuroprotection in stroke: the importance of collaboration and reproducibility. Brain 140, 2079–2092. 10.1093/brain/awx126 28641383

[B22] Niu F. Song X. Y. Hu J. F. Zuo W. Kong L. L. Wang X. F. (2017). IMM-H004, a new coumarin derivative, improved focal cerebral ischemia via blood-brain barrier protection in rats. J. Stroke Cerebrovasc. Dis. 26, 2065–2073. 10.1016/j.jstrokecerebrovasdis.2016.11.121 28669655

[B23] Peisker T. Koznar B. Stetkarova I. Widimsky P. (2017). Acute stroke therapy: a review. Trends Cardiovasc. Med. 27, 59–66. 10.1016/j.tcm.2016.06.009 27471112

[B24] Peña I. D. Borlongan C. Shen G. Davis W. (2017). Strategies to extend thrombolytic time window for ischemic stroke treatment: an unmet clinical need. J. Stroke 19, 50–60. 10.5853/jos.2016.01515 28178410 PMC5307939

[B25] Rowland A. Miners J. O. Mackenzie P. I. (2013). The UDP-glucuronosyltransferases: their role in drug metabolism and detoxification. Int. J. Biochem. Cell Biol. 45, 1121–1132. 10.1016/j.biocel.2013.02.019 23500526

[B26] Shan L. Yang S. Zhang G. Zhou D. Qiu Z. Tian L. (2014). Comparison of the inhibitory potential of bavachalcone and corylin against UDP-glucuronosyltransferases. Evid. Based Complement. Alternat. Med. 2014, 958937. 10.1155/2014/958937 24829606 PMC4009204

[B27] Shirai M. Yamanishi R. Moon J. H. Murota K. Terao J. (2002). Effect of quercetin and its conjugated metabolite on the hydrogen peroxide-induced intracellular production of reactive oxygen species in mouse fibroblasts. Biosci. Biotechnol. Biochem. 66, 1015–1021. 10.1271/bbb.66.1015 12092810

[B28] Song X. Y. Hu J. F. Sun M. N. Li Z. P. Wu D. H. Ji H. J. (2013). IMM-H004, a novel coumarin derivative compound, protects against amyloid beta-induced neurotoxicity through a mitochondrial-dependent pathway. Neuroscience 242, 28–38. 10.1016/j.neuroscience.2013.02.049 23523945

[B29] Song X. Y. Hu J. F. Sun M. N. Li Z. P. Zhu Z. X. Song L. K. (2014). IMM-H004, a novel coumarin derivative compound, attenuates the production of inflammatory mediatory mediators in lipopolysaccharide-activated BV2 microglia. Brain Res. Bull. 106, 30–38. 10.1016/j.brainresbull.2014.05.002 24878446

[B30] Stain-Texier F. Boschi G. Sandouk P. Scherrmann J. M. (1999). Elevated concentrations of morphine 6-beta-D-glucuronide in brain extracellular fluid despite low blood-brain barrier permeability. Br. J. Pharmacol. 128, 917–924. 10.1038/sj.bjp.0702873 10556926 PMC1571713

[B31] Subramaniam S. R. Ellis E. M. (2013). Neuroprotective effects of umbelliferone and esculetin in a mouse model of Parkinson’s disease. J. Neurosci. Res. 91, 453–461. 10.1002/jnr.23164 23184853

[B32] Sulakhiya K. Keshavlal G. P. Bezbaruah B. B. Dwivedi S. Gurjar S. S. Munde N. (2016). Lipopolysaccharide induced anxiety- and depressive-like behaviour in mice are prevented by chronic pre-treatment of esculetin. Neurosci. Lett. 611, 106–111. 10.1016/j.neulet.2015.11.031 26620836

[B33] Sun M. Hu J. Song X. Wu D. Kong L. Sun Y. (2013). Coumarin derivatives protect against ischemic brain injury in rats. Eur. J. Med. Chem. 67, 39–53. 10.1016/j.ejmech.2013.04.015 23835481

[B34] Tourancheau A. Rouleau M. Guauque-Olarte S. Villeneuve L. Gilbert I. Droit A. (2018). Quantitative profiling of the UGT transcriptome in human drug-metabolizing tissues. Pharmacogenomics J. 18, 251–261. 10.1038/tpj.2017.5 28440341

[B35] Uchaipichat V. Mackenzie P. I. Guo X. H. Gardner-Stephen D. Galetin A. Houston J. B. (2004). Human UDP-glucuronosyltransferases: isoform selectivity and kinetics of 4-methylumbelliferone and 1-naphthol glucuronidation, effects of organic solvents, and inhibition by diclofenac and probenecid. Drug Metab. Dispos. 32, 413–423. 10.1124/dmd.32.4.413 15039294

[B36] Vaidyanathan J. B. Walle T. (2002). Glucuronidation and sulfation of the tea flavonoid (-)-epicatechin by the human and rat enzymes. Drug Metab. Dispos. 30, 897–903. 10.1124/dmd.30.8.897 12124307

[B37] Wang Q. Ye C. Jia R. Owen A. J. Hidalgo I. J. Li J. (2006). Inter-species comparison of 7-hydroxycoumarin glucuronidation and sulfation in liver S9 fractions. In Vitro Cell. Dev. Biol. Anim. 42, 8–12. 10.1007/s11626-006-0004-z 16618213

[B38] Wang X. Li R. Wang X. Fu Q. Ma S. (2015). Umbelliferone ameliorates cerebral ischemia-reperfusion injury via upregulating the PPAR gamma expression and suppressing TXNIP/NLRP3 inflammasome. Neurosci. Lett. 600, 182–187. 10.1016/j.neulet.2015.06.016 26071904

[B39] Xia Y. L. Liang S. C. Zhu L. L. Ge G. B. He G. Y. Ning J. (2014). Identification and characterization of human UDP-glucuronosyltransferases responsible for the glucuronidation of fraxetin. Drug Metab. Pharmacokinet. 29, 135–140. 10.2133/dmpk.DMPK-13-RG-059 24025985

[B40] Yamazaki S. Miyoshi N. Kawabata K. Yasuda M. Shimoi K. (2014). Quercetin-3-O-glucuronide inhibits noradrenaline-promoted invasion of MDA-MB-231 human breast cancer cells by blocking b2-adrenergic signaling. Arch. Biochem. Biophys. 557, 18–27. 10.1016/j.abb.2014.05.030 24929186

[B41] Yang P. F. Song X. Y. Zeng T. Ai Q. D. Liu D. D. Zuo W. (2017). IMM-H004, a coumarin derivative, attenuated brain ischemia/reperfusion injuries and subsequent inflammation in spontaneously hypertensive rats through inhibition of VCAM-1. RSC Adv. 7, 27480–27495. 10.1039/C7RA02154B

[B42] Zhang Z. Wu X. Zhao M. Yang Y. Wang Y. Hu J. (2017). Determination of IMM-H004, a novel neuroprotective agent, in rat plasma and brain tissue by liquid chromatography–tandem mass spectrometry. J. Chromatogr. B Analyt. Technol. Biomed. Life Sci. 1048, 49–55. 10.1016/j.jchromb.2017.02.005 28213295

[B43] Zuo W. Chen J. Zhang S. Tang J. Liu H. Zhang D. (2014). IMM-H004 prevents toxicity induced by delayed treatment of tPA in a rat model of focal cerebral ischemia involving PKA-and PI3K-dependent Akt activation. Eur. J. Neurosci. 39, 2107–2118. 10.1111/ejn.12551 24649933

[B44] Zuo W. Zhang W. Han N. Chen N. H. (2015). Compound IMM-H004, a novel coumarin derivative, protects against CA1 cell loss and spatial learning impairments resulting from transient global ischemia. CNS Neurosci. Ther. 21, 280–288. 10.1111/cns.12364 25601434 PMC6495400

